# Relationship of severity of hyponatremia and adverse outcomes in children visiting the emergency department

**DOI:** 10.3389/fped.2024.1379727

**Published:** 2024-06-14

**Authors:** Jisu Ryoo, Arum Choi, Hyunchul Cho, Woori Bae

**Affiliations:** ^1^ Department of Emergency Medicine, College of Medicine, The Catholic University of Korea, Seoul, Republic of Korea; ^2^ Department of Radiology, College of Medicine, The Catholic University of Korea, Seoul, Republic of Korea; ^3^ Medical Service Corps of 11th Aviation Group, Republic of Korea Army, Namyangju, Republic of Korea

**Keywords:** hyponatremia, child, adverse outcome, pediatric emergency department, mortality

## Abstract

Mild hyponatremia is often found in patients visiting pediatric emergency departments (PEDs), but there are few large-scale studies on its association with adverse outcomes, including mortality. We conducted this study to identify the association of mild hyponatremia with adverse outcome. This retrospective observational study included children under 18 years of age visiting the PED at a tertiary hospital. We used electronic medical record data from January 1, 2009 to December 31, 2020. Clinical outcomes, including ward admission, vasopressor administration, pediatric intensive care unit (PICU) admission, and mortality, were assessed for the total of 44,147 patients. Among these, 1,639 (3.7%) were in the hyponatremia group, with 1,521 (3.4%) exhibiting mild hyponatremia. Mild hyponatremia was more prevalent in younger patients, particularly in the 1–3 years age group, and less common in females. Patients with mild hyponatremia had a significantly prolonged median length of stay in the PED compared to normonatremic patients (5.8 h vs. 4.4 h, *p* < 0.001). Moreover, they showed significantly higher rates of ward admission (51.1% vs. 35.6%, *p* < 0.001), vasopressor administration (1.1% vs. 0.6%, *p* = 0.014), PICU admission (2.4% vs. 1.0%, *p* < 0.001), and mortality (1.5% vs. 0.3%, *p* < 0.001). Compared with the normonatremia group, the odds ratios (95% CI) for ward admission, vasopressor administration, PICU admission, and mortality in the mild hyponatremia group were 1.90 (1.71–2.10), 1.91 (1.17–3.13), 2.62 (1.86–3.68), and 5.56 (3.51–8.80), respectively. Furthermore, our findings demonstrate a notable upward trend in adverse outcomes, including vasopressor administration, PICU admission, and mortality, from mild hyponatremia to severe hyponatremia. In conclusion, we found that adverse outcomes increase with the severity of hyponatremia in children presenting to the PED, highlighting the importance of immediate intervention alongside the identification of the underlying cause.

## Introduction

Hyponatremia is the most common electrolyte disorder and is defined as a serum sodium concentration <135 mEq/L (1). It typically occurs with excess intake of water and a reduced capacity to excess free water. Under normal circumstances, the body attempts to prevent hyponatremia by producing diluted urine to eliminate excess free water (2).

Hyponatremia in the pediatric emergency department (PED) is mainly related to inflammation and infections, such as pneumonia, meningitis, and urinary tract infection (3). Excessive sweating, poor oral intake, vomiting, and diarrhea due to infection can lead to volume depletion and hypovolemic hyponatremia (4). Proinflammatory cytokines released during inflammation stimulate the secretion of vasopressin, causing euvolemic hyponatremia (3). Vasopressin helps regulate water balance in the body by increasing water reabsorption in the kidneys, which can cause water retention and potentially contribute to conditions such as hyponatremia when excessively secreted (3).

Although hyponatremia is usually straightforward to diagnose and rarely life-threatening, it raises several concerns. One key concern is that if left untreated or mismanaged, hyponatremia can lead to potentially life-threatening complications such as cerebral edema, seizures, and respiratory distress (1). Furthermore, compromised ability to regulate electrolyte levels during illness can exacerbate sodium imbalance. Additionally, in critically ill patients, hyponatremia can complicate the overall clinical outcomes. In one study, patients with hyponatremia showed a higher risk of underlying infection, hospital admission, and hospital stay lasting more than 5 days than individuals with normonatremia (5). Moreover, when compared to normonatremia, lower sodium levels were linked to a 60% longer length of stay (LOS) in the pediatric intensive care unit (PICU) and a two-fold increase in complications within the PICU (5). In another study, children with hyponatremia experienced a relatively elevated mortality rate, which reached as high as 20% (6).

Hyponatremia can lead to adverse patient outcomes. Although the relationship between the presence of hyponatremia and severity of infection has been well-documented, a few studies have shown an association between mild hyponatremia and adverse outcomes in PED settings (7, 8). Therefore, this study aimed to investigate the relationship between severity of hyponatremia and adverse outcomes in children visiting the PED. We also assessed the tendency for adverse outcomes based on the severity of hyponatremia.

## Materials and methods

### Study design and setting

This single-center retrospective study undertook in the PED of a tertiary hospital. Data were obtained from January 1, 2009, to December 31, 2020.

### Participants

This study included patients aged <18 years who visited a PED. Patients lacking blood test results, diagnostic codes, or records of disposition, those with hypernatremia, and patients with serum glucose levels above 250 mg/dl were excluded from the study (9).

### Data source

From the electronic medical records of eligible patients throughout the study duration, we extracted the following variables: age, gender, blood sodium levels, arrival mode, diagnoses recorded in the PED, PED LOS, vasopressors administration in the PED, admission to the general ward, admission to the PICU, and mortality rate. Diagnoses were categorized on the basis of Korean Standard Classification of Diseases 7 (KCD-7) codes, including gastrointestinal, respiratory, neurologic, infection, hemato-oncologic, trauma, genitourinary, cardiac, and others (10).

### Definition of hyponatremia

Hyponatremia was defined as a serum sodium concentration of <135 mmol/L and was further categorized as mild (130–134 mEq/L), moderate (129–125 mEq/L), or severe (<125 mEq/L).

### Outcomes

We conducted a comprehensive analysis to assess various adverse outcomes, including vasopressor administration, ward admission, PICU admission, and mortality, in relation to mild hyponatremia and normonatremia observed during the initial PED visit. We also assessed changes in the rate of adverse outcomes based on patients' sodium concentration.

### Statistical analysis

Differences between groups were assessed using specific tests based on the nature of the variables. Categorical variables were analyzed using the chi-square test, and continuous variables were analyzed using the Mann-Whitney test.

Odds ratios (ORs) were calculated to evaluate the relationship between sodium concentration and dependent variables. These calculations were performed with a 95% confidence interval (CI) using binary logistic regression analysis. The analysis was adjusted for variables such as age, sex, arrival mode, and diagnosis.

All statistical analyses were performed using R version 4.0.0, developed by the R Foundation for Statistical Computing, Vienna, Austria. The significance level was set at *p* < 0.05.

### Ethical considerations

This study was carried out in adherence with the Declaration of Helsinki and received approval by the Institutional Review Board (IRB) of the Catholic University of Korea (IRB approval no. KC21RASI0238).

## Results

**Figure 1:**
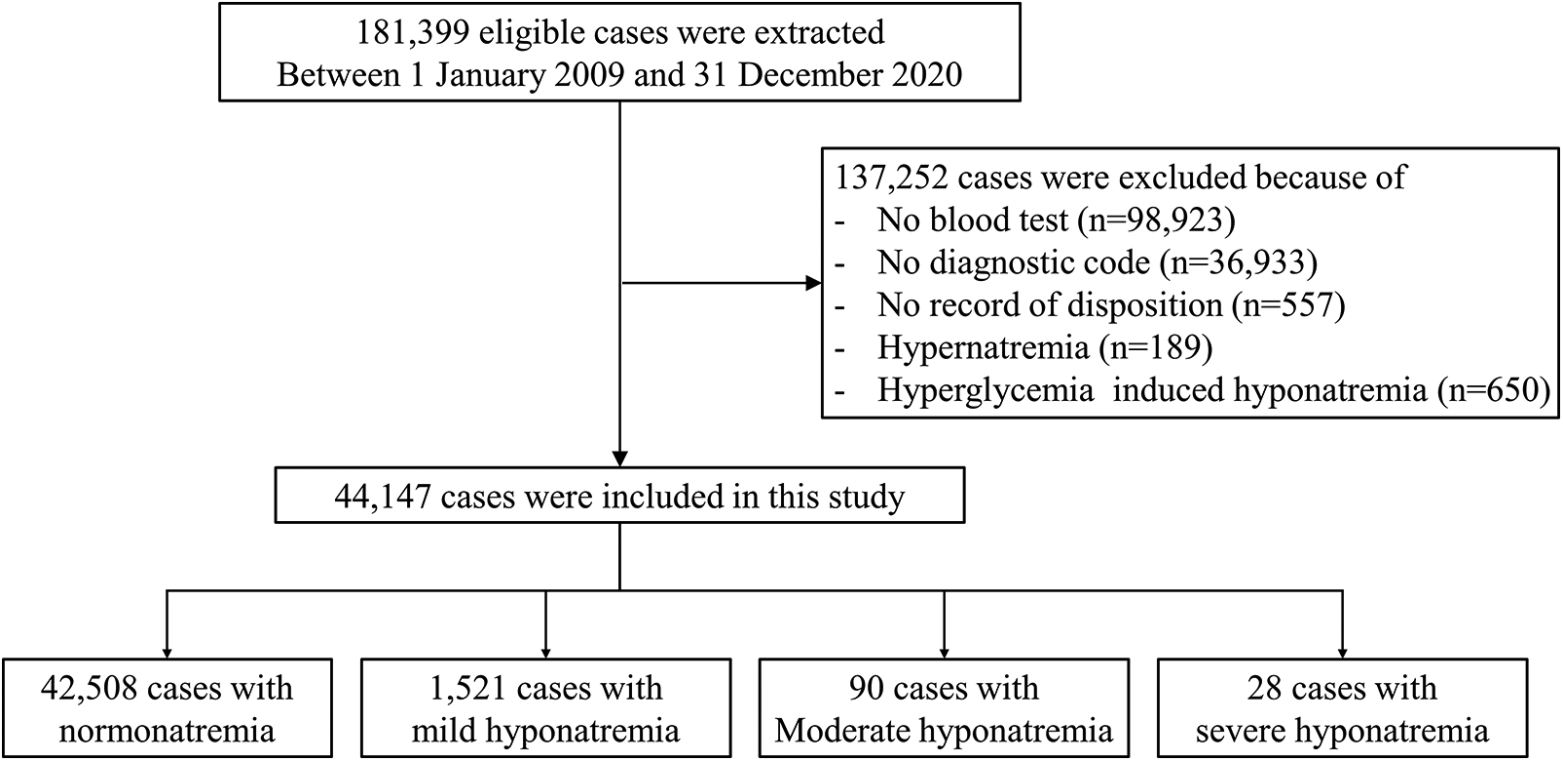
Flowchart of the study population.

Between January 2009 and December 2020, 181,399 patients sought medical care in the PED at a tertiary hospital. Exclusions comprised 98,923 patients without blood testing, 36,933 patients lacking diagnostic codes, 557 individuals without a recorded disposition, 189 patients with hypernatremia, and 650 patients with high serum glucose levels. Finally, 44,147 patients were involved in this study (Figure 1).

Within this cohort, 1,639 patients (3.7%) were categorized into the hyponatremia group, of which 1,521 (3.4%) showed mild hyponatremia. When comparing patients with mild hyponatremia to those with normal sodium levels, the median age in the mild hyponatremia group (48 mos) was significantly lower than that in the normonatremia group (61 mos; *p* < 0.001). Notably, the mild hyponatremia group showed a higher proportion of patients in the 1–3-year-old and 4–6-year-old age groups, while the 0–12-month-old, 7–12-year-old, and 13–17-year-old age groups were less represented in the mild hyponatremia group than in the normonatremia group. Additionally, the proportion of females in the mild hyponatremia group was significantly lower (38.2% vs. 45.0%; *p* < 0.001). The most prevalent diagnoses in both the mild hyponatremia and normonatremia groups, listed in descending order of frequency, were respiratory, infectious, and hemato-oncological diseases (Table 1).

**Table 1:** Characteristics of patients (*n* = 44,029).

Variables	Mild hyponatremia 130 ≤ Na ≤ 134 (*n* = 1,521)	Normonatremia 135 ≤ Na ≤ 145 (*n* = 42,508)	*P*
Age (month), median (IQR)	48.0 (23.0–94.0)	61.0 (23.0–141.0)	<0.001
0–12months	134 (8.8%)	5,490 (12.9%)	<0.001
1–3years	621 (40.8%)	12,642 (29.7%)	
4–6years	337 (22.2%)	7,074 (16.6%)	
7–12years	265 (17.4%)	8,243 (19.4%)	
13–17years	164 (10.8%)	9,059 (21.3%)	
Female sex	581 (38.2%)	19,106 (45.0%)	<0.001
Vital sign at presentation, median (IQR)			
Heart rate	114.0 (100.0–130.0)	108.0 (96.0–122.0)	<0.001
Respiration rate	22.0 (20.0–24.0)	22.0 (20.0–24.0)	<0.001
Body temperature	38.0 (37.1–39.0)	37.3 (36.7–38.2)	<0.001
Diagnosis			
Respiratory	458 (30.1%)	13,128 (30.9%)	<0.001
Gastrointestinal	72 (4.7%)	3,180 (7.5%)	
Hemato-oncologic	229 (15.1%)	4,254 (10.0%)	
Neurologic	112 (7.4%)	2,298 (5.4%)	
Trauma	18 (1.2%)	1,866 (4.4%)	
Infectious	457 (30.1%)	11,838 (27.9%)	
Genitourinary	25 (1.6%)	1,135 (2.7%)	
Cardiac	8 (0.5%)	353 (0.8%)	
Others	142 (9.3%)	4,456 (10.5%)	
Mode of arrival			
Self-referred	1,106 (72.8%)	32,197 (75.8%)	<0.001
Outpatient department	176 (11.6%)	3,640 (8.6%)	
Referred from clinic	238 (15.7%)	6,666 (15.7%)	

Values are presented as *n* (%) unless otherwise indicated.

A significant difference in the median PED LOS was observed among the mild hyponatremia and normonatremia groups. Specifically, the mild hyponatremia group showed a median LOS of 5.8 h, which was significantly longer than that in the normonatremia group (4.4 h; *p* < 0.001). Moreover, the mild hyponatremia group exhibited significantly higher rates of adverse outcomes than the normonatremia group. The ward-admission rate in the mild hyponatremia group was 51.1%, a figure surpassing the 35.6% observed in the normonatremia group (*p* < 0.001). Similarly, the rates of vasopressor administration and PICU for the mild hyponatremia group were 1.1% and 2.4%, respectively, which were notably elevated compared to corresponding rates in the normonatremia group (0.6% and 0.9%, respectively; *p* = 0.014 and *p* < 0.001, respectively). In addition, the mild hyponatremia group showed a significantly higher mortality rate (1.5%) than the normonatremia group, with a mortality rate of 0.3% (*p* < 0.001; Table 2).

**Table 2:** Outcomes of patients (*n* = 44,029).

Variable	Mild hyponatremia 130 ≤ Na ≤ 134 (*n* = 1,521)	Normonatremia 135 ≤ Na ≤ 145 (*n* = 42,508)	*P*
PED Length of stay (h), median (IQR)	5.8 (3.9–11.3)	4.4 (3.0–7.5)	<0.001
Ward admission	777 (51.1%)	15,114 (35.6%)	<0.001
Use of vasopressor during PED	17 (1.1%)	250 (0.6%)	0.014
PICU admission	37 (2.4%)	401 (0.9%)	<0.001
Mortality	22 (1.5%)	112 (0.3%)	<0.001

Values are presented as *n* (%) unless otherwise indicated.

PED, pediatric emergency department; PICU, pediatric intensive care unit.

**Figure 2:**
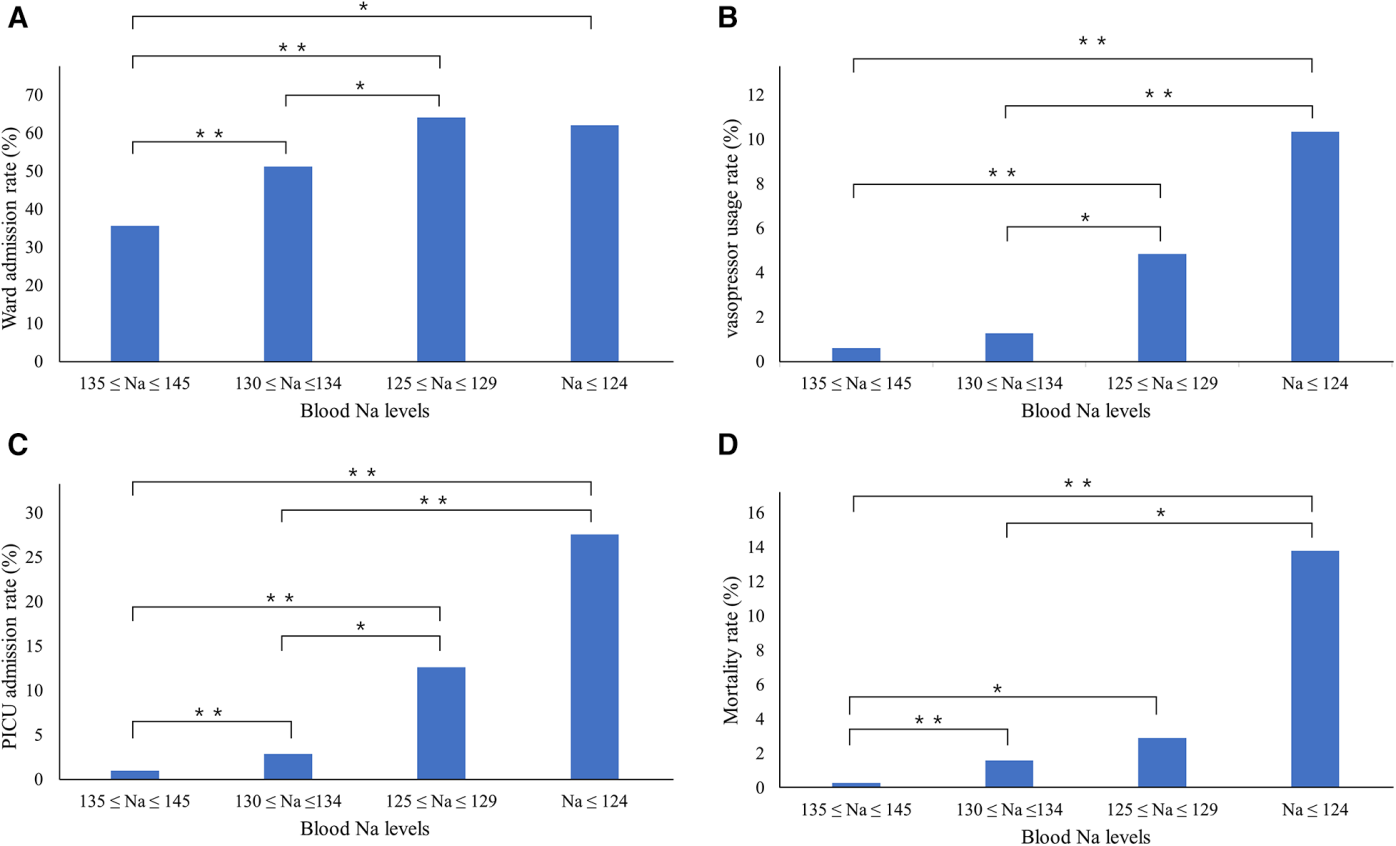
Changes in adverse outcome rates in relation to serum sodium concentrations. The (**A**) ward admission rates, (**B**) vasopressor use rates, (**C**) PICU admission rates, and (**D**) mortality rates increase significantly with increasing serum sodium concentrations. One asterisk (*) indicates *p*-value smaller than 0.05 (*p* < 0.05). Two asterisks (**) indicate *p*-value smaller than 0.01 (*p* < 0.01). PICU, pediatric intensive care unit.

Adverse outcomes were assessed on the basis of the serum sodium concentration by categorizing the patients into four groups: normonatremia, mild hyponatremia, moderate hyponatremia, and severe hyponatremia. Notably, the frequency of vasopressor administration and PICU admission showed a statistically significant upward trend from the normonatremia group to the severe hyponatremia group. In the PED, the rates of vasopressor admission were 0.6%, 1.1%, 5.6%, and 10.7% (*p *< 0.001), respectively, while the rates of PICU admission were 0.9%, 2.4%, 8.9%, and 25.0% (*p *< 0.001), respectively. Furthermore, the mortality rates increased significantly across groups (0.3%, 1.5%, 3.3%, and 14.3%, respectively; *p *< 0.001). However, ward admissions showed a different pattern, progressively increasing from mild to moderate hyponatremia but decreasing for patients with severe hyponatremia. For the mild, moderate, and severe hyponatremia groups, the ward-admission rates were 51.1%, 66.7%, and 64.3%, respectively (*p* < 0.001; Figure 2).

In comparison with the normonatremia group, the OR with 95% CI for adverse outcomes, including vasopressor admission, ward admission, PICU admission, and mortality, are provided in Table 3.

**Table 3:** Logistic regression analysis of patient outcomes (*n* = 44,147).

Variable	Normonatremia 135 ≤ Na ≤ 145 (*n* = 42,508)	Hyponatremia (*n* = 1,639)		
		Mild hyponatremia 130 ≤ Na ≤ 134 (*n* = 1,521)	Moderate hyponatremia 125 ≤ Na ≤ 129 (*n* = 90)	Severe hyponatremia Na ≤ 124 (*n* = 28)
Ward admission	1 (reference)	1.90 (1.71–2.10)	3.63 (2.34–5.62)	3.26 (1.51–7.07)
*p*-value		<0.001	<0.001	0.003
Use of vasopressor during PED	1 (reference)	1.91 (1.17–3.13)	9.94 (4.00–24.71)	20.28 (6.09–67.61)
*p*-value		0.01	<0.001	<0.001
PICU admission	1 (reference)	2.62 (1.86–3.68)	10.24 (4.92–21.31)	35.00 (14.80–82.80)
*p*-value		<0.001	<0.001	<0.001
Mortality	1 (reference)	5.56 (3.51–8.80)	13.05 (4.07–41.88)	63.09 (21.54–184.78)
*p*-value		<0.001	<0.001	<0.001

Values are presented as odds ratio (95% confidence interval).

Logistic regression analysis was adjusted for age, gender, diagnosis, and mode of arrival.

PED, pediatric emergency department; PICU, pediatric intensive care unit.

## Discussion

This study revealed that mild hyponatremia has a significant effect on pediatric patient outcomes in PED settings, including vasopressor administration, ward admission, PICU admission, and mortality.

First, our demographic analysis revealed a higher prevalence of mild hyponatremia in younger age groups, particularly among children aged 1–3 years. This observation was similar but slightly different from that of a previous study, in which hyponatremia was most common among those aged <12 mos (63%) (11). In a previous study, upper respiratory diseases and infections were the leading causes of PED visits in children aged 1–3 years, with the highest incidence rates in this age group (12). Thus, the prevalence of upper respiratory diseases and infections in younger populations may play a role in the development of hyponatremia. Additionally, our analysis revealed a significantly lower proportion of females in the mild hyponatremia group, which is supported by a cross-sectional study reporting an OR of 1.36 for male sex in a comparison of hyponatremia with normonatremia (7). These demographic differences emphasize the potential influence of age- and sex-related factors in the development of hyponatremia.

One of the most compelling findings was the extended median LOS in the PED for patients with mild hyponatremia. The mild hyponatremia and normonatremia groups exhibited median LOS values of 5.8 and 4.4 h, respectively. This prolonged PED LOS in patients with mild hyponatremia implies a more complex clinical course and the need for additional medical attention, and underscores the clinical significance of mild hyponatremia and the challenges it presents in the PED setting. Our findings align with those of a previous study, in which patients with mild hyponatremia who had hospital stays exceeding 5 days showed a significantly higher OR of 1.79 in comparison with patients with normonatremia who also had prolonged hospital stays (13).

Adverse outcomes were more frequent in the mild hyponatremia group than in the normonatremia group, including significantly higher rates of vasopressor administration, ward admission, PICU admission, and mortality. Notably, the ward-admission rate for patients with mild hyponatremia was 51.1%, which significantly exceeded the rate of 35.6% in the normonatremia group. Thus, mild hyponatremia was associated with a higher likelihood of requiring inpatient care. Similarly, the rates of vasopressor administration and PICU admission were notably higher in the mild hyponatremia group, suggesting that even mild hyponatremia can complicate the clinical course and lead to more severe outcomes. A previous study showed that adult patients with hyponatremia have higher rates of ICU admissions and were more frequently admitted to post-acute care facilities (14). Another study reported that mild hyponatremia is related to a higher risk of hospital admission (adjusted OR, 1.72; 95% CI, 1.06–2.48) (13).

The most striking difference was observed in the mortality rates. The mild hyponatremia group exhibited a significantly higher mortality rate of 1.5%, contrasting with the 0.3% mortality rate in the normonatremia group. This substantial difference highlights the potential life-threatening consequences of mild hyponatremia in children, underscoring the importance of not underestimating this condition. Consistent with a study involving hospitalized adults, our findings showed that individuals with an admission serum sodium concentration ranging from 130 to 134 mEq/L had a 24% higher risk of mortality 5 years after admission than those with normonatremia (*p *< 0.001) (15). On the basis of these findings, we suggest that even mild hyponatremia should not be overlooked, but detected and corrected early in the PED setting.

Furthermore, an in-depth analysis of adverse outcomes based on the severity of hyponatremia revealed a clear upward trend. As the severity of hyponatremia increased, the likelihoods of vasopressor use, PICU admission, and mortality increased significantly. These findings align with the results of previous studies. In a study conducted by Arzu et al. involving children admitted to the PICU, a clear association was established between the severity of hyponatremia, increased use of vasoactive drugs, and mortality (16). Similar results were reported in a study of hospitalized adult patients, in which mortality increased proportionally with the severity of hyponatremia (17). This underscores the importance of recognizing the severity of hyponatremia as a critical factor in assessing the risk of adverse outcomes in the PED setting, demonstrating that even mild hyponatremia can lead to poor prognosis. In contrast, while the rate of ward admissions increased progressively from mild to moderate hyponatremia, it decreased in patients with severe hyponatremia. This is likely due to the fact that children with severe hyponatremia are either immediately admitted to the PICU or die while in the PED.

The relationship between hyponatremia and these adverse outcomes can be explained by various mechanisms. Hyponatremia is often associated with inflammatory conditions and infections, primarily due to the non-osmotic release of vasopressin, an antidiuretic hormone. Proinflammatory cytokines like tumor necrosis factor-α, IL-1β, and IL-6, especially IL-6, play central roles in the release of vasopressin (18). During active inflammation, these cytokines can independently or synergistically stimulate the hypothalamus-pituitary-adrenal axis, leading to vasopressin release, even in the absence of traditional triggers such as changes in serum osmolality. Increased prostaglandin E2 and nitric oxide production during active inflammation causes downregulation of the epithelial sodium channel and Na/K ATPase gene expression, thereby downregulating sodium transport. A previous study found that high C-reactive protein levels were associated with low serum sodium concentrations in children with sepsis and malignancy (19). Another study suggested that hyponatremia occurs in patients with Kawasaki disease and severe inflammation (20). Furthermore, another study suggested that hyponatremia could be an important inflammatory marker in pediatric febrile urinary tract infection because it is associated with the degree of inflammation (21). As reported previously, hyponatremia in febrile illness indicates more severe infection and inflammation. Therefore, patients with hyponatremia in a PED setting may have more severe conditions than patients with normonatremia, resulting in more frequent adverse outcomes. Additionally, the complications of hyponatremia may contribute to adverse outcomes. Hyponatremia causes several complications such as cerebral edema. Children face a heightened risk of symptomatic hyponatremia and tend to develop hyponatremic encephalopathy at higher serum sodium levels than adults (22). Several studies consistently recognize hyponatremia as an independent predictor of mortality, adjusting for parameters reflecting the severity of an underlying disease (23). Mortality is linked to mild hyponatremia as well, regardless of the underlying disease (15).

This study's principal strength lies in its comprehensive analysis of a large dataset over a decade from a single tertiary hospital's PED. By examining a substantial number of children, this study provides strong insights into the impact of hyponatremia on various clinical outcomes in the PED setting. Additionally, the inclusion of a wide range of outcome measures, such as vasopressor administration, ward admission, PICU admission, and mortality, enhances the breadth and depth of the findings.

This study has some limitations. First, it employed a retrospective design and analyzed data extracted from the electronic medical records of a single institution. Therefore, the findings may not be easily generalizable to all PED visits. Nevertheless, this limitation was partially mitigated by the use of a substantial dataset spanning over a decade for our analysis. Second, due to the anonymous nature of the data in this study, the potential inclusion of information from the same patient on multiple occasions could not be ruled out. However, this limitation is inherent in studies that use anonymized data and is not unique to this study.

Future research will employ more robust statistical methods to adjust for potential confounders and biases, thereby enhancing the reliability of our findings regarding the relationship between hyponatremia and adverse outcomes. Moreover, comprehensive analyses of patient data will be conducted with the objective of identifying potential physiological markers associated with severe outcomes. This will provide a more comprehensive understanding of the mechanisms by which hyponatremia contributes to higher mortality rates.

To enhance the generalizability of our findings, future studies will stratify patients by age and specific diagnostic groups. This stratification will permit a more detailed examination of the impact of hyponatremia across various patient demographics, thereby enhancing the applicability of our results to clinical practice.

## Conclusion

Mild hyponatremia in children presenting to the PED is related to adverse outcomes such as vasopressor administration, ward admission, PICU admission, and mortality. Furthermore, our findings indicate that an increase in the severity of hyponatremia is associated with a negative impact on the various outcomes. Therefore, when mild hyponatremia is identified in a pediatric patient visiting the PED, immediate intervention should be implemented in addition to identifying the cause. These interventions are crucial for improving adverse outcomes and patient care in the emergency department setting.

## Data Availability

The raw data supporting the conclusions of this article will be made available by the authors, without undue reservation.
